# Distribution, composition and functions of gelatinous tissues in deep-sea fishes

**DOI:** 10.1098/rsos.171063

**Published:** 2017-12-06

**Authors:** Mackenzie E. Gerringer, Jeffrey C. Drazen, Thomas D. Linley, Adam P. Summers, Alan J. Jamieson, Paul H. Yancey

**Affiliations:** 1 Department of Oceanography, University of Hawai‘i at Mānoa, 1000 Pope Road, Honolulu, HI 96822, USA; 2 Oceanlab, University of Aberdeen, Main Street, Newburgh, Aberdeenshire AB41 6AA, UK; 3 Friday Harbor Labs, University of Washington, Friday Harbor, WA 98250, USA; 4 Biology Department, Whitman College, Walla Walla, WA 99362, USA

**Keywords:** subdermal extracellular matrix, buoyancy, Liparidae, hadal, swimming biomechanics, robotic model

## Abstract

Many deep-sea fishes have a gelatinous layer, or subdermal extracellular matrix, below the skin or around the spine. We document the distribution of gelatinous tissues across fish families (approx. 200 species in ten orders), then review and investigate their composition and function. Gelatinous tissues from nine species were analysed for water content (96.53 ± 1.78% s.d.), ionic composition, osmolality, protein (0.39 ± 0.23%), lipid (0.69 ± 0.56%) and carbohydrate (0.61 ± 0.28%). Results suggest that gelatinous tissues are mostly extracellular fluid, which may allow animals to grow inexpensively. Further, almost all gelatinous tissues floated in cold seawater, thus their lower density than seawater may contribute to buoyancy in some species. We also propose a new hypothesis: gelatinous tissues, which are inexpensive to grow, may sometimes be a method to increase swimming efficiency by fairing the transition from trunk to tail. Such a layer is particularly prominent in hadal snailfishes (Liparidae); therefore, a robotic snailfish model was designed and constructed to analyse the influence of gelatinous tissues on locomotory performance. The model swam faster with a watery layer, representing gelatinous tissue, around the tail than without. Results suggest that the tissues may, in addition to providing buoyancy and low-cost growth, aid deep-sea fish locomotion.

## 1. Introduction

**Figure 1.**
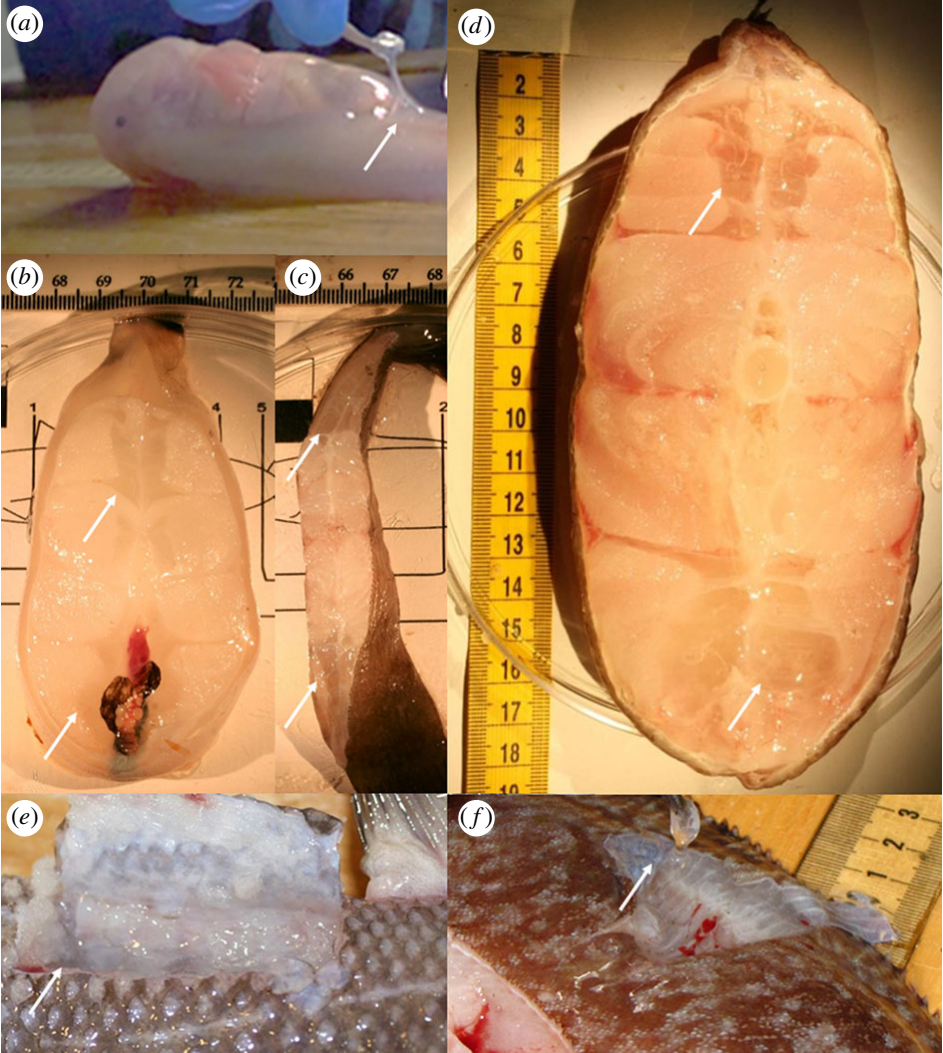
Gelatinous tissues. Arrows point to gelatinous tissue layers. (*a*) *Notoliparis kermadecensis*, family Liparidae, hadal snailfish. Gelatinous tissues prominent directly below skin, concentrated around posterior of cavity and along tail. *Photo by J. Reed. Image courtesy of the HADES Program, NSF, NOAA OER, (© WHOI).* (*b*–*d*) Cross sections of fishes showing gelatinous tissues bundles. (*b*) Twoline eelpout, *Bothracara brunneum,* family Zoarcidae. (*c*) Deep-sea sole, *E. bathybius*, family Pleuronectidae. *Photos by J. Friedman.* (*d*) Giant cusk eel, *Spectrunculus grandis*, family Ophidiidae. *Photo by P. Yancey.* (*e*) Gelatinous tissues between muscle bands in *Coryphaenoides yaquinae*, family Macrouridae. *Photo by M. Gerringer.* (*f*) *Embassichthys bathybius* gelatinous tissues, within musculature and lifted by scalpel. *Photo by P. Yancey*.

In some species of ray-finned fishes (Actinopterygii), a distinct watery tissue layer is present, usually between the skin and muscle or between muscle bundles (figure 1). Fishes in the superorder Elopomorpha (Anguilliformes, Albuliformes, Elopiformes and Saccopharyngiformes) have larvae called leptocephali in which most of the body consists of an acellular gelatinous matrix that provides structural support in the absence of a vertebral column and transparency for camouflage (e.g. [1,2]). The first known scientific record of these tissues in a fully adult fish comes from the Challenger Report description of the gelatinous blind cusk eel *Aphyonus gelatinosus*, in which the ‘anterior half of the skin forms a large loose bag which, during life, is probably filled and distended with mucus' [3]. Gelatinous tissue is even a defining character in the genus *Careproctus* of the family Liparidae (snailfish), which ‘best illustrates the production of pseudotissue which envelops the body and fins just beneath the skin’ [4]. The tissues are sometimes referred to as the subdermal extracellular matrix, or SECM (e.g. [5,6]). More recently, such tissues have been found in hadal snailfishes in the Kermadec and Mariana trenches. In a freshly collected fish, the layer of clear gelatinous tissue is prominent (figure 1*a*), although as the skin is lacerated, this tissue leaks out and melts away. It is largely concentrated just behind the abdominal cavity, with a thin layer around the posterior third of the body.

Although these gelatinous tissues have been noted in several deep-living adult species and can compose up to a third of the mass of a fish [5], they have not been compared across families and their functions remain unresolved. In addition to structural support and transparency, one possible role proposed for gelatinous larval fishes (e.g. [7]) and some deep-sea invertebrates (e.g. [8]) is to allow growth to large size at low metabolic cost. This hypothesis may apply to adult fishes as well. One study investigated the potential antifreeze function of the gelatinous tissues in an Antarctic fish, but found no evidence to suggest a role in cold-tolerance [9]. Eastman *et al*. [5] found free nerve endings present within the gelatinous tissues of *Paraliparis devriesi*. It was hypothesized that these may serve as mechanoreceptors in three Antarctic liparids, allowing the fish to detect displacement of the gelatinous layer during movement [10,11]. The potential sensory role of gelatinous tissues, however, is proposed to be secondary to another function—buoyancy.

Gelatinous layers have been described in a number of mid-water fishes, leading to the hypothesis that they are an adaptation for buoyancy, first introduced by Denton & Marshall [12] and expanded by Davenport & Kjorsvik [13] and Yancey *et al*. [14]. In all but the deepest-living teleost fishes, internal ion concentrations and osmolalities are lower than seawater. For example, extracellular fluids of typical shallow teleosts have about 170 mM NaCl and lesser amounts of other ions, yielding an osmolality of 350–400 mOsm kg^−1^ (e.g. [15]). In comparison, average seawater has roughly 500 mM NaCl plus other ions yielding about 1000–1100 mOsm kg^−1^. Thus, extracellular fluid, including that in gelatinous tissues, with very little non-lipid organic material will be less dense than seawater (unlike many tissues such as muscle, bone and cartilage). In addition, some gelatinous tissues in mid-water fishes have even lower ion concentrations than other body fluids, increasing buoyancy even more [14]. The buoyancy hypothesis was further supported by Eastman *et al*. [5] in a study of gelatinous tissues in the Antarctic snailfish, *P. devriesi*, which are believed to achieve neutral buoyancy through decreased bone ossification and the presence of this layer. These low-density tissues and fluids would be adaptive under the high hydrostatic pressures of the deep sea, where the inflation of a swimbladder becomes increasingly difficult [16].

Gelatinous tissues could also act as fairing along the fish's tail, creating a better hydrofoil and improved swimming efficiency, especially in liparids and aphyonids. Davenport & Kjorsvik [13] touched on this idea briefly, suggesting that there may be an exoskeletal function to gelatinous tissue in *Cyclopterus lumpus*. They note that the gelatinous tissue was more prominent in females than males, up to 18% of body mass, and show that the males used more high-amplitude tail beats to swim than females. Our results suggest that this may be a much more broadly used strategy. Support for this concept is inferred from studies of tadpole swimming, where a ‘fish-shaped’ body required significantly less power to swim than a ‘tadpole-shaped’ body [17]. The same authors later found that the tadpole morphology creates form drag where the tail meets the body, resulting in the decreased swimming efficiency [18]. The tadpole shape is selected against in pond experiments where fish predators are present, further illustrating the advantage to losing those high drag zones [19]. The location of the gelatinous tissue within the hadal snailfishes, concentrated around the anterior of the body cavity and under the skin along the tail, suggests that it could act to counteract this effect. An optimization model of body shape in fishes showed the wide head and tapered tail to be an efficient shape for undulatory swimming [20]. We propose that the gelatinous tissues could allow the fish to reach this streamlined shape without producing more muscle, reducing the need for the high-amplitude, energetically expensive tail beats required of tadpole-shaped forms [18].

References to the presence and function of gelatinous tissues have often been speculative and passing. Here, we analyse compositions of these tissues in selected species, evaluate the proposed buoyancy function, synthesize and review references to gelatinous tissues, investigate depth-related trends in the presence of these tissues and introduce a new hypothesis: gelatinous tissues may be an adaptive method of changing body shape at low growth cost, acting as a fairing material to increase locomotor performance.

## 2. Material and methods

### 2.1. Proximate chemistry and buoyancy tests

*Samples.* Fishes were collected by otter trawl from Monterey Bay in April and October 2009 (details by [21]) and by baited trap in the Kermadec Trench in 2011 and 2014. Collection information for gelatinous tissues analysed in this study is presented in electronic supplementary material, table S1. *Buoyancy.* Fresh pieces of gelatinous and white muscle tissues were placed at mid-depth in a graduated cylinder or glass jar filled with seawater at 2–5°C shortly after capture, and sink or rise times (to travel 6 cm) were measured. *Water content.* Gelatinous tissues were dried at 60°C for 3 days to ensure that all water evaporated and remaining dry mass was compared to original wet tissues mass. *Osmotic pressure.* A vapour pressure osmometer, Wescor 5500, was used in the laboratory for most species, and at sea for *Notoliparis kermadecensis*, to determine sample osmolality. Samples were homogenized with a small pestle in a microfuge tube, then centrifuged at 2000 × *g* for 30 min at 4°C. Ten microlitres of the resulting supernatant was measured with an osmometer. The 290 and 1000 mmol kg^−1^ standards were checked periodically to confirm accurate calibration. *Sample preparation.* A section of frozen gelatinous tissues, clear of white muscle, was cut and weighed to obtain about 0.1 g, with a precision of 0.0001 g. The section was ground in 7% perchloric acid (PCA) or 70% ethanol, added at nine times the tissues mass, to precipitate proteins. The sample was refrigerated overnight, then centrifuged for 20 min at 15 500 × *g* at 4°C. The supernatant, transferred to a new tube, was used for inorganic ion and organic osmolyte analyses, while the pellet was used for protein analysis. When ethanol was used to homogenize tissues, the supernatant was evaporated and the remaining powder dissolved in distilled water. The supernatants in PCA were titrated with 2 M KOH to pH 6.5–7.5. The resulting precipitate was centrifuged and the supernatant removed to a new tube. The PCA method was not used for ion analysis because of the required addition of potassium. *Protein.* Protein content was determined with the bicinchoninic acid protein assay [22]. Bovine serum albumin was used as a standard. *Lipids.* Lipid contents were analysed using the Bligh & Dyer [23] extraction and colorimetric determination of content with the sulfuric acid charring method of Marsh & Weinstein [24] with triolein as a standard. *Carbohydrates.* Carbohydrate analysis was conducted using phenol and sulfuric acid [25], with d-glucose as a standard, measured in a spectrophotometer (Beckman Coulter DU 730) at 480 nm. *Ions.* Sodium and potassium contents were analysed by atomic absorption (PerkinElmer AAnalyst 400) in 10 µl aliquots of the PCA homogenates dissolved in 10 ml of purified water. All results are presented as average ± standard deviation.

### 2.2. Taxonomic distribution

Records of gelatinous tissues in fishes were collected in an extensive literature search. Recent findings from coastal to hadal surveys are also presented. Anecdotally, these tissues were thought to be more common in deeper-living fishes. To test this, common depth ranges of fishes with gelatinous tissues were taken from FishBase [26]. Care was taken to avoid records that were obviously spurious or outlying, for example, several thousand metres out of all other capture and sighting records. The effects of phylogenetic relationships can confound interpretation of this type of analysis, as closely related species become a kind of pseudoreplicate [27]. To account for this potential error, and to clarify the distribution of gelatinous tissues, we compared depth trends within clades. Statistical analyses were conducted in the programming platform R [28]. Generalized linear models (GLM) using minimum and maximum depths and the median of each depth range were fitted using a Gaussian error distribution. Models were selected through optimization of Akaike information criteria.

### 2.3. Locomotor effects

**Figure 2.**
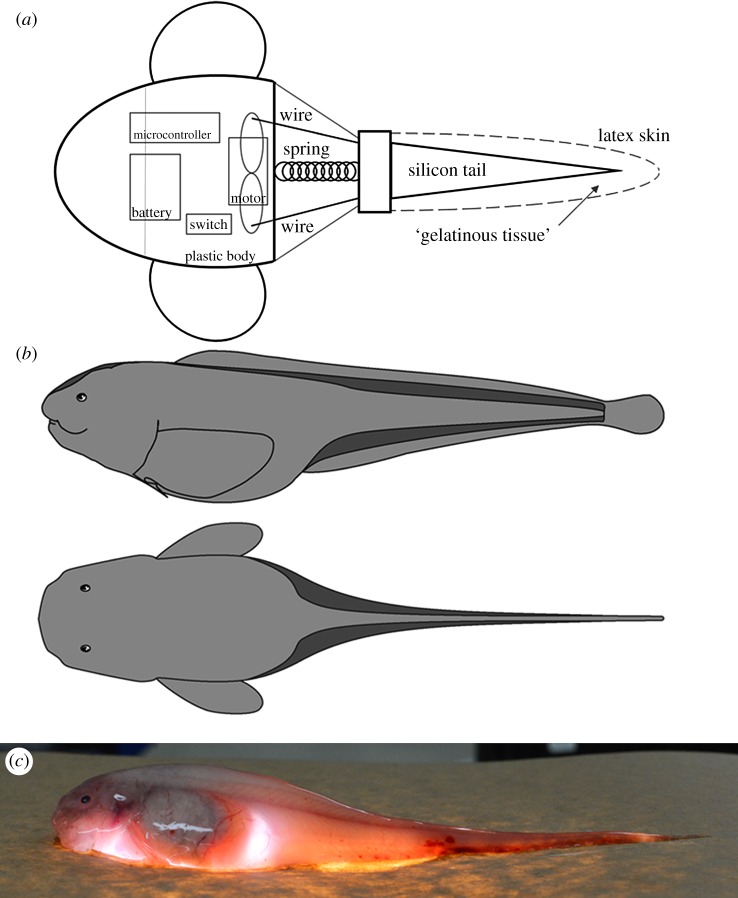
(*a*) Schematic of robotic hadal snailfish model. Microcontroller (Arduino Nano), motor (Tower Pro TM, Micro Servo 9 g, SG90), battery (Duracell, 9 V). Tail muscle is a cast silicone rubber (Ecoflex R 00-10) with a volume-adjustable skin (latex condom, Trojan Magnum). Additional materials used include hot glue, a spring, piano wire, a bottle cap, marine epoxy, electrical tape and miscellaneous hardware as ballast. Dotted line indicates outer skin, kept empty in trials with no gelatinous tissue analogue. (*b*) Hadal liparid body shape with gelatinous tissues in dark grey. Dorsal and anal fin rays connect to epaxial and hypaxial muscle tissue while gelatinous tissues surround. *Drawing by T. Linley. (c*) Hadal liparid *N. kermadecensis* on illuminated platform, highlighting gelatinous tissues. *Photo by J. Reed. Image courtesy of the HADES Program, NSF, NOAA OER, (© WHOI)*.

Few studies have investigated locomotion in deep-sea fishes (e.g. [29–32]), largely due to the difficulty of direct experimentation. To test the effect of body shape change with gelatinous tissues, a robotic model was designed after the Kermadec Trench snailfish, *N. kermadecensis*, a good example of a neutrally buoyant species with large amounts of gelatinous tissues. This technique has become a valuable tool used to investigate swimming biomechanics in a number of shallow-living fishes (e.g. [33–35]) and is well-suited to deep-sea species that cannot easily be brought into a laboratory setting. The plastic (polylactic acid) body and fins were three-dimensional printed (ORION HB #58744) based on a model constructed from a photogrammetry recreation of freshly captured specimens collected on the HADES (HADal Ecosystems Studies) Cruise in April and May of 2014 (Model: MeshMixer, Slicing: Cura, 3D Printing: Repetier Host). The free-swimming, neutrally buoyant robotic model was larger (40 cm SL) than the actual hadal snailfish (known maximum 29 cm SL) due to design constraints. The model motion program was controlled by an on-board Arduino Nano microcontroller. Tail-beat frequency (0.5 Hz) was chosen to match that found through video analysis of the hadal snailfish, *Pseudoliparis belyaevi*, filmed *in situ* in the Japan Trench (described in [36]). The robot was powered by a 9 V battery with constant cycle-averaged power and swam using a servomotor connected to two piano wires that oscillated the tail region back and forth (figure 2). A silicone rubber mould was cast to simulate the posterior skeleton and musculature of the fish. Water between the silicon tail analogue and outer skin represented the gelatinous tissues, to isolate the shape effect from changes due to tail stiffness. The model was designed to test the locomotor effects of gelatinous tissues that are directly below the skin, outside of the muscle tissue, such as in the hadal snailfish. As discussed, this positioning is not consistent across taxa and the locomotor effects may vary accordingly. In some species, such as the cusk eel *Spectrunculus grandis*, it is unlikely that the gelatinous tissue flows freely as water in our model would. However, in the liparids, morphological analyses suggest that gelatinous layers are displaced during movement [10,11]. This is also suggested by video of hadal snailfishes swimming *in situ*, which show the gelatinous tissues rippling under the skin, making water below the skin, rather than gelatine, an appropriate analogue. Two approximately 10 s swim trials for the submerged, neutrally buoyant robot were conducted with both empty and full tail ‘skin’. Swim trials were filmed from above as the robot swam in a 1 m diameter tank, and body lengths per second and tail-beat amplitude were compared between trials (with the same tail-beat frequency and power) using ImageJ [37].

## 3. Results

### 3.1. Buoyancy and proximate chemistry

In shipboard buoyancy experiments, gelatinous tissues from most species floated in seawater, the only exception being tissues from *N. kermadecensis*, which appeared to be neutrally buoyant (did not rise or sink in the cylinder). When placed in cold (2°C) seawater, a whole hadal snailfish sank very slowly, tail first. Float rates were collected for gelatinous tissues from five species. Tissues travelled 6 cm upwards in 2.96 ± 0.26 s (*Bothracara brunneum*, *n* = 4), 2.53 ± 0.86 s (*Embassichthys bathybius*, *n* = 9), 3.55 ± 0.60 s (*Microstomus pacificus*, *n* = 3), 1.16 ± 0.31 s (*P. karenae*, *n* = 3) and 3.71 ± 0.80 s (*S. grandis* 2000 m, *n* = 4).

Analyses of nine species (common depths 750–7500 m) revealed that tissues were primarily water (96.5 ± 1.8%) with minor amounts of other constituents (table 1). Protein, carbohydrate and lipid contents were low (0.39 ± 0.23, 0.61 ± 0.28, and 0.69 ± 0.57, respectively). Sodium contents were much higher than potassium contents (Na : K ratio from 18 to 38; Welch two-sample *t*-test, *p* ≤ 0.0001), as is typical of extra- but not intracellular fluids. Sodium contents also trended higher with depth (157 mmol kg^−1^ at 1000 m to 362 at 7000 m) both inter- and intraspecifically (e.g. *S. grandis*, 205 mmol kg^−1^ at 2000 versus 318 mmol kg^−1^ at 4149 m). Most tissues had similar potassium contents (6.5–12.8 mmol kg^−1^), though higher in the deepest fish, *N. kermadecensis* (14.4 ± 0.7 mmol kg^−1^). Osmolalities, in mOsm kg^−1^, were measured in gelatinous tissues of six species. Values ranged from 311 to 385 in four species from 1000 to 2000 m, and were higher in the two deeper species analysed, most notably *N. kermadecensis* at 945 mOsm kg^−1^.

**Table 1.** Proximate chemistry of gelatinous tissues in representative species. Numbers in parentheses indicate sample size for each analysis. Capture depth in metres. Sodium, potassium given in mmol kg^−1^ wet mass and osmolality in mOsm kg^−1^. *Bothracara brunneum* osmolality value from 2000 m sample.

species	capture depth	potassium	sodium	Na/K	% water	% protein	% carb	% lipid	osmolality
*Careproctus melanurus*	750–1000	8.47 ± 0.82 (3)	157 ± 30.4 (3)	18.5	98.4 ± 0.26 (3)	0.21 ± 0.22 (3)	0.99 (1)	0.2 (1)	
*Careproctus cypselurus*	1000	8.51 (1)	158 (1)	18.6	97.9 (1)	0.23 (1)	0.51 (1)	0.15 (1)	
*Embassichthys bathybius*	1000	7.24 ± 2.5 (4)	187 ± 23.8 (4)	25.9	97.0 ± 1.32 (4)	0.25 ± 0.09 (4)	0.51 ± 0.19 (4)	1.58 ± 1.77 (3)	377 ± 16.2 (3)
*Microstomus pacificus*	1000	8.33 ± 3.24 (3)	188 ± 5.27 (3)	22.5	96.4 ± 1.24 (3)	1.1 ± 1.15 (3)	0.54 ± 0.2 (3)	0.97 ± 0.73 (3)	312 (1)
*Bothrocara brunneum*	1000–2000	9.23 ± 1.24 (2)	196 ± 7.42 (2)	21.2	97.6 ± 0.84 (3)	0.37 ± 0.03 (3)	0.58 (1)	0.28 ± 0.19 (2)	385 (1)
*Spectrunculus grandis*	2000	12.8 (1)	205 (1)	16.0	96.5 (1)	0.63 (1)	1.25 (1)		355 (1)
*Pachycara karenae*	3000	6.54 ± 0.47 (3)	195 ± 14.5 (3)	29.9	95.8 ± 1.13 (3)	0.65 ± 0.28 (3)	0.38 (1)	1.31 ± 0.12 (2)	467 (1)
*Spectrunculus grandis*	4149	8.31 (1)	318 (1)	38.3					
*Pyrolycus* sp.	4817	8.20 (1)	284 (1)	34.6					
*Notoliparis kermadecensis*	7000–7500	14.4 ± 0.72 (3)	362 ± 38.4 (3)	28.4	93.1 ± 0.55 (3)	0.65 ± 0.09 (3)			945 ± 78.7 (5)

### 3.2. Taxonomic distribution

**Figure 3.**
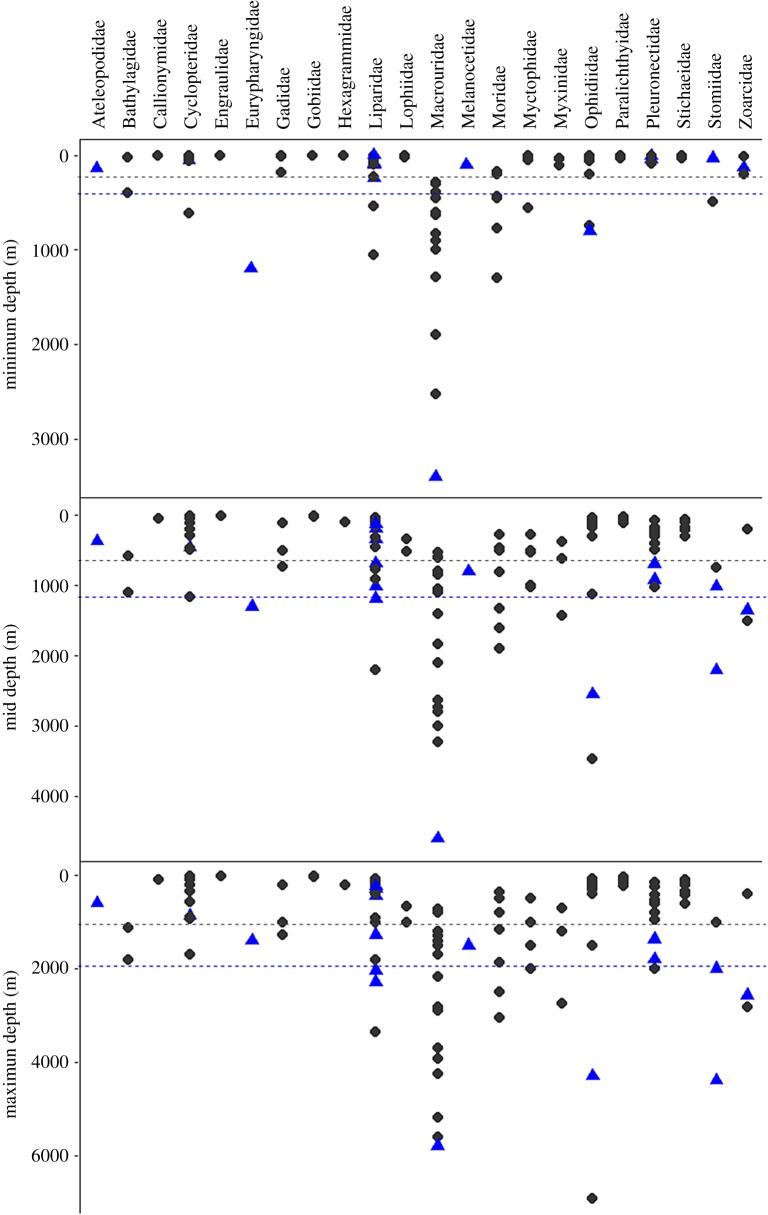
Depth ranges of species with and without gelatinous tissues compared in the present study. Species with gelatinous tissues shown in blue triangles, those without gelatinous tissues grey circles. Grouped by family. Average depths of species with (blue) and without (grey) gelatinous tissues shown as dotted line.

Fish species with gelatinous tissues were found in ten orders, thirteen families and approximately 200 species, presented in table 2. References to ‘gelatinous tissues’ or ‘subdermal extracellular matrix’ were included in these results. Fishes in the family Aphyonidae (recently absorbed into the Bythitidae; [42]) are described, for example: ‘skin loose, transparent and gelatinous' [44]. Images of freshly caught fish confirm that this refers to the subdermal gelatinous tissues. Other occurrences of gelatinous tissues have been seen and verified by the authors in recent captures. We note that the gelatinous tissues are present in many, but not all, species of the snailfish genus *Paraliparis*. Additional species of the genus *Lycodapus* may contain gelatinous tissues as well, though this has not been confirmed [7]. Depth ranges for species with gelatinous tissues are presented in table 2. Median depths of occurrence ranged from approximately 300 to 7400 m (mean approximately 1800 m). Most species with records of gelatinous tissues typically live around or below 1000 m depth and include both benthic and pelagic species. GLM showed fishes with gelatinous tissues to have significantly deeper minimum, median and maximum depths (*t* = 2.40, *p* < 0.05; *t* = 3.01, *p* < 0.01; *t* = 2.95, *p* < 0.01; 117 d.f.) across all species, a finding confirmed by a non-parametric Kruskal–Wallis rank sum test (median and maximum depths, *p* ≤ 0.01, *p* = 0.001) across all species. This was also a significant trend within orders (e.g. Gadiformes, minimum: *t* = 6.70, *p* < 0.005, median: *t* = 4.75, *p* < 0.005, maximum: *t* = 3.20, *p* < 0.01, 27 d.f.; Pleuronectiformes, median: *t* = 3.0, *p* < 0.01, maximum: *t* = 3.1, *p* < 0.01, 16 d.f.). Species with gelatinous tissues were present across multiple clades, and represent the deeper-living species within clades (figure 3).

**Table 2.** Fishes with gelatinous tissues, from literature and current capture data. Reference indicates the publication that describes the gelatinous tissues. Larval fishes with gelatinous tissues not included.

order	family	genus or species	depth range	reference
Ateleopodiformes	Ateleopodidae	*Ateleopus japonicus*	140–600 [38]	[6]
Gadiformes	Macrouridae	*Coryphaenoides yaquinae*	3400–6945 [39,40]	(present study)
Lophiiformes	Melanocetidae	*Melanocetus johnsonii*	100–1500 [41]	(present study)
Ophidiiformes	Bythitidae	23 species*	2000–6000 [43]	[44]
	Ophidiidae	*Apagesoma delosommatus*	2487–4150 [44]	[44]
		*Apagesoma edentatum*	5082–8082 [44]	[44]
		*Barathrites iris*	?–5285 [45]	(present study)
		*Spectrunculus grandis*	800–4300 [46]	(present study)
Osmeriformes	Bathylagidae	*Bathylagus pacificus*	772–7700** [47]	[14]
		*Pseudobathylagus milleri*	772–6600** [48]	[14]
Perciformes	Zoarcidae	*Bothrocara brunneum*	129–2570 [49]	(present study)
		*Derepodichthys alepidotus*	1000–2904 [50]	[51]
		*Lycodapus mandibularis*	100–1370 [52]	[53]
		*Pachycara karenae*	2780–3100 [54]	(present study)
Pleuronectiformes	Pleuronectidae	*Embassichthys bathybius*	41–1800 [47]	[55]
		*Microstomus pacificus*	10–1370 [47]	[56]
Saccopharyngiformes	Eurypharyngidae	*Eurypharynx pelecanoides*	1200–1400 [57]	[12]
Scorpaeniformes	Cyclopteridae	*Cyclopterus lumpus*	0–868 [58]	[13]
	Liparidae	*Careproctus,* 119 species	6– > 5000 [59]	[60--62]
		*Lipariscus nanus*	0–910 [47]	[63]
		*Nectoliparis pelagicus*	557–3383 [49]	[63]
		*Notoliparis,*4 species	5879–7669 [64]	(present study)
		*Paraliparis,* 26 species	233–2150 [65,66]	[5,11,65--67]
		*Psednos balushkini*	914–917 [67]	[67]
		*Psednos gelatinosus*	0–650 [68]	[68]
		*Psednos nataliae*	1100–1120 [67]	[67]
		*Pseudoliparis,* 3 species	6198–8098 [36,70]	[69,70]
Stomiiformes	Stomiidae	*Chauliodus macouni*	25–4390 [71]	[14]
		*Chauliodus sloani*	494–1000 [48]	[12]
		*Tactostoma macropus*	30–2000 [48]	[14]

*Those species formerly classified as family Aphyonidae [42].

**Pelagic fish, maximum depth greatly overestimated [36]. Note that some, but not all species of the genus *Paraliparis* have gelatinous tissues. Depth ranges presented in metres.

### 3.3. Locomotor effects

Gelatinous tissues change the body shape of the hadal liparid, as illustrated in figure 2. Intuitively, this changes the drag profile around the animal. In all swim trials, the robotic model performed significantly better with the gelatinous tissue analogue (0.074 ± 0.007 body lengths per second) than without (0.022 ± 0.007) with constant cycle-averaged power provided at a constant tail-beat frequency of 0.5 Hz (Welch two-sample *t*-test, *p* = 0.019). Tail-beat amplitude was 16.1 ± 0.3% of body length and did not vary significantly between treatments (*p* > 0.05). Films of swim trials are provided in the electronic supplementary material. Additional tests were conducted in the harbour to ensure that wall effects were not confounding swim trials, yielding similar results (data not shown).

## 4. Discussion

### 4.1. Distribution and composition

Proximate chemical analysis of gelatinous tissues in nine benthic and benthopelagic species showed high water content and low protein, lipid and carbohydrate content in comparison to white muscle (86.3 ± 2.7% for seven species with gelatinous tissues; [21]). Our average for water content (96.5%) is consistent with previous studies of gelatinous tissues, which found 93.3% water in *C. lumpus* [13], 96% in *Bathylagus pacificus* [14] and 97% in *P. devriesi* [5]. Osmolality increases with depth, in part due to higher extracellular sodium and in part because of organic osmolytes (especially trimethylamine oxide) that increase with depth to combat the negative effects of high hydrostatic pressure [64,72]. In concert with the ionic concentrations and osmolalities of these tissues, these data suggest that the layers are of similar compositions and are mainly extracellular fluid. The high seawater content of gelatinous tissues makes them inexpensive to produce in bulk.

We found support for the hypothesis that gelatinous tissues in fishes are a characteristically deep-sea phenomenon. Phylogenetic relationships were a potential concern; especially as there are certain genera where gelatinous tissues are more common—i.e. *Aphyonus, Careproctus, Paraliparis*. The method of Felsenstein [27] for investigating trends without confounding influence of phylogeny is designed for use with continuous variables, but has been met with criticism for categorical variables (e.g. [73]). Considering these concerns, we investigated depth trends within clades. The results hold true within the different orders tested: gelatinous tissues appear more often in deeper-living species.

Records of gelatinous tissues were found across multiple orders and families (table 2). In several recent phylogenetic hypotheses of major groups within the Actinopterygii based on multi-locus molecular datasets, gelatinous tissues are present in species across many clades, from basal to highly derived [74,75]. The fact that the gelatinous tissues are present across ten orders suggests the potential for the independent development of SECM tissue in multiple deep-water groups. It is possible that these tissues have evolved from different origins, given their varied locations in different species—such as directly under the skin or closer to the spine (figure 1).

Although we were thorough in our literature searches and covered a broad depth range in our surveys, it is likely that the list presented in table 2 is not exhaustive. Often, the tissue has leaked away shortly after capture or during preservation, and it is not always recorded in taxonomic descriptions or it is regarded as unimportant. This study reveals how common and multi-functional gelatinous tissues may be, and we suggest that future studies should note its presence.

### 4.2. Gelatinous tissues as a buoyancy mechanism

In our investigation, most gelatinous tissues did float in shipboard tests, suggesting that buoyancy is indeed a main function of these tissues, in agreement with most previous findings [13,14]. The one exception was the deepest fish tested, *N. kermadecensis* (hadal snailfish), which also had a significantly higher potassium content and lower per cent water than other species (table 2), indicating more intracellular components than in other species. These buoyancy and composition results suggest that the gelatinous tissue is not positively buoyant in that species. It is possible that testing at atmospheric pressure may have biased these results because these fish were collected from considerably greater depths than the other species. Observations of the swimming behaviour of these fish *in situ* suggest that the entire fish is slightly negatively buoyant, settling to the seabed when active swimming ceases. This swimming behaviour has been observed in multiple hadal trench liparids (*N. kermadecensis, Pseudoliparis amblystomopsis*, *P. swirei*). These fishes do not have swim bladders, and the gelatinous tissues, even if not positively buoyant, would have lower density than most other tissues (e.g. bone, muscle), so may help reduce overall body density and thus rate of sinking, as previously suggested for *Chauliodus sloani*, a pelagic viperfish species that also has gelatinous tissue that is not positively buoyant [12]. Additionally, the gelatinous SECM is often found in fishes with aglomerular kidneys and lacking gas bladders, such as *Ateleopus japonicus*, which may serve to reduce whole-animal density [6]. It is possible that aglomerular kidneys result in increased water retention and thus the accumulation of gelatinous tissues. However, the correlation between the aglomerular kidney and gelatinous tissues remains to be fully explored.

As noted earlier, previous work on mesopelagic fishes revealed lower ion concentrations in gelatinous tissue compared to blood [14]. Our osmolality values hint at a similar pattern for gelatinous tissue because they are well below osmolalities of blood and muscle of other fish species from comparable depths. Muscle osmolalities at 1000 m have been reported at approximately 400 (cf. gelatinous tissues at 312–377), at 2000 m approximately 490–500 (cf. gelatinous tissues at 355–385), at 3000 m approximately 590–600 (cf. gelatinous tissues at 467) and at 7000 m approximately 990–1000 (cf. gelatinous tissues at 945, [64,72]). It should be noted that there would be an energetic cost associated with actively maintaining the ionic gradient needed to produce greater buoyancy (e.g. ion-regulating chloride cells in gelatinous tissues of leptocephali, [76]).

While most of the gelatinous tissues tested could aid fish buoyancy, our results suggest that this might not be the only function. Importantly, gelatinous tissues are found in some species with gas-filled swim bladders (e.g. family Ophidiidae), indicating that buoyancy may not always be their primary adaptive role. Furthermore, gelatinous tissues are found in benthic flatfishes (order Pleuronectiformes; e.g. *E. bathybius* and *M. pacificus*), which would have less evolutionary pressure to develop positively buoyant tissues, as they spend more time resting on the seafloor than swimming. In several species, gelatinous tissues are concentrated ventrally, an unlikely position to provide positive buoyancy. Some bathypelagic fishes are within 0.5 and 1.2% (*Gonostoma elongatum* and *Xenodermichthys copei*) of neutral buoyancy without swim bladders or gelatinous layers, through reduced ossification and watery muscle tissue [12] and some benthopelagic fishes lacking gas bladders also have watery muscle to aid in achieving neutral buoyancy [77]. In the hadal liparids, near neutral buoyancy seems also maintained by other means, including a large fatty liver and reduced bone ossification.

### 4.3. Locomotor effects

Watery gelatinous tissues may be used to increase body size at lower production cost than muscle tissue, a strategy noted earlier that has been proposed for some deep-sea invertebrates (e.g. [8]) and some larval fishes (e.g. [7]). Gelatinous tissues may be an example of neoteny, where deep-sea species may have evolved to retain this low-growth-cost paedomorphic character into adulthood in a food-poor environment. In the hadal snailfish, *N. kermadecensis*, there does seem to be more gelatinous tissue in larger individuals, although the exact amount of tissue could not be quantified due to damage. Some deep-sea fishes, including two flatfish in this study, also have very watery muscle tissue, which further reduce growth costs, though, in this case, by sacrificing locomotory capacity [77]. The gelatinous tissues are the extreme end of this continuum. They serve as low-growth-cost bulk tissues, allowing the animal to grow large, reducing the likelihood of predation, without alteration to locomotory muscle.

Material properties of the actual gelatinous tissues should also be analysed under deep-sea, especially hadal, temperatures and pressures, as even small changes in body shape and stiffness can make a large difference in swimming performance (e.g. [33,78]). Gelatinous tissues (which melt at room temperature) are probably stiffer at hadal conditions of cold temperatures and high pressures, and could provide an even better paddle for forward propulsion. There may be an additional cost of transport to the stiffer tail, though this may improve acceleration [79]. Gelatinous tissues may change stiffness and shape with movement, as seen in other models of undulatory swimming (e.g. [80]). While further exploration of this hypothesis is needed, the improved performance of the robotic model with a gelatinous tissue analogue suggests that the presence of a subdermal gelatinous layer could enhance swimming performance. The chemical composition of the gelatinous tissues shows that they are inexpensive to form, but the benefit to structure and locomotory capacity could be significant, accounting for some of its prevalence across many deep-sea genera. However, this use of gelatinous tissue cannot be universal; when the gelatinous tissue occurs within the main musculature of a fish (e.g. figure 1*b*) no locomotory advantage is likely.

## 5. Conclusion

Our results suggest that gelatinous tissues are widely used by fishes, principally in deep-sea species, serving multifunctional roles both for individual fish and across families. Gelatinous tissues, which are primarily extracellular fluid, are present in fishes of very different life histories and behaviours, from the flatfish, *M. pacificus*, to the hadal snailfish, *N. kermadecensis*. The varied location of gelatinous tissues, which are present in the trunk of some eelpouts (Zoarcidae), the snout of *Ateleopus japonicas* (Ateleopodidae) and directly below the skin in many snailfishes (Liparidae), also calls attention to potential functional complexity. Through chemical analyses and float tests, we found support for the use of gelatinous tissues in aiding fish buoyancy. Robotic modelling supported the hypothesis that these tissues may also provide a functional role in reducing drag during swimming. Overall, gelatinous tissues seem to be a low-density, low-production-cost method to increase body size and alter body shape and size, with adaptive advantages for both swimming efficiency and buoyancy with varied functions among species.

## Supplementary Material

Supplementary Table 1

## Supplementary Material

Supplementary Table 2

## Data accessibility

The datasets supporting this article have been uploaded as part of the electronic supplementary material.

## References

[1] Pfeiler E 1999 Developmental physiology of elopomorph leptocephali. Comp. Biochem. Physiol. 123A, 113–128. (doi:10.1016/S1095-6433(99)00028-8)

[2] Miller MJ 2009 Ecology of anguilliform leptocephali: remarkable transparent fish larvae of the ocean surface layer. Aqua-BioSci. Monogr. 2, 1–94. (doi:10.5047/absm.2009.00204.0001)

[3] Günther A 1887 Report on the deep-sea fishes collected by H. M. S. Challenger during the years 1873--76. Chall. Rep. 22, 335 pp.

[4] Burke V 1930 Revision of fishes of family Liparidae. *Bulletin of the United States National Museum*, **150**, i--xii, 1--204.

[5] Eastman J, Hikida R, Devries A 1994 Buoyancy studies and microscopy of skin and subdermal extracellular matrix of the Antarctic snailfish, *Paraliparis devriesi*. J. Morphol. 220, 85–101. (doi:10.1002/jmor.1052200108) 10.1002/jmor.1052200108 29865380

[6] Ozaka C, Yamamoto N, Somiya H 2009 The aglomerular kidney of the deep-sea fish, *Ateleopus japonicus* (Ateleopodiformes: Ateleopodidae): evidence of wider occurrence of the aglomerular condition in Teleostei. Copeia 2009, 609–617. (doi:10.1643/CP-08-131)

[7] Marliave J, Peden A 1989 Larvae of *Liparis fucensis* and *Liparis callyodon*: is the ‘cottoid bubblemorph’ phylogenetically significant? Fish. Bull. 87, 735–743.

[8] Mitra A, Zaman S 2016 Basics of marine and estuarine ecology, 481 p New Delhi, India: Springer India.

[9] Jung A, Johnson P, Eastman J, Devries A 1995 Protein content and freezing avoidance properties of the subdermal extracellular matrix and serum of the Antarctic snailfish, *Paraliparis devriesi*. Fish Physiol. Biochem. 14, 71–80. (doi:10.1007/BF00004292) 24197273 10.1007/BF00004292

[10] Eastman JT, Lannoo MJ 1998 Morphology of the brain and sense organs in the snailfish *Paraliparis devriesi*: neural convergence and sensory compensation on the Antarctic shelf. J. Morphol. 237, 213–236. (doi:10.1002/(SICI)1097-4687(199809)237:3<213::AID-JMOR2>3.0.CO;2-#) 9734067 10.1002/(SICI)1097-4687(199809)237:3<213::AID-JMOR2>3.0.CO;2-#

[11] Lannoo MJ, Eastman JT, Orr JW 2009 Nervous and sensory systems in sub-Arctic and Antarctic snailfishes of the genus *Paraliparis* (Teleostei: Scorpaeniformes: Liparidae). Copeia 2009, 732–739. (doi:10.1643/CG-08-157)

[12] Denton EJ, Marshall NB 1958 The buoyancy of bathypelagic fishes without a gas-filled swim bladder. J. Mar. Biol. Assoc. 37, 753–767.

[13] Davenport J, Kjorsvik E 1986 Buoyancy of the lumpsucker *Cyclopterus lumpus*. J. Mar. Biol. Assoc. 66, 159–174. (doi:10.1017/S0025315400039722)

[14] Yancey P, Lawrence-Berrey R, Douglas M 1989 Adaptations in mesopelagic fishes. Mar. Biol. 103, 453–459. (doi:10.1007/BF00399577)

[15] Prosser C, Mackay W, Kato K 1970 Osmotic and ionic concentrations in some Alaskan fish and goldfish from different temperatures. Physiol. Zool. 43, 81–89. (doi:10.1086/physzool.43.2.30155517)

[16] Scholander PF 1954 Secretion of gases against high pressures in the swimbladder of deep sea fishes. Biol. Bull. 107, 260–277. (doi:10.2307/1538612)

[17] Liu H, Wassersug R, Kawachi K 1996 A computational fluid dynamics study of tadpole swimming. J. Exp. Biol. 199, 1245–1260. 9319105 10.1242/jeb.199.6.1245

[18] Liu H, Wassersug R, Kawachi K 1997 The three-dimensional hydrodynamics of tadpole locomotion. J. Exp. Biol. 200, 2807–2819. 9344964 10.1242/jeb.200.22.2807

[19] Johnson JB, Saenz D, Adams CK, Hibbitts TJ 2015 Naturally occurring variation in tadpole morphology and performance linked to predator regime. Ecol. Evol. 5, 2991–3002. (doi:10.1002/ece3.1538) 26357533 10.1002/ece3.1538 PMC4559044

[20] Eloy C 2013 On the best design for undulatory swimming. J. Fluid Mech. 717, 48–89. (doi:10.1017/jfm.2012.561)

[21] Drazen JC, Friedman JR, Condon NE, Aus EJ, Gerringer ME, Keller AA, Clarke EM 2015 Enzyme activities of demersal fishes from the shelf to the abyssal plain. Deep Sea Res. Part I Oceanogr. Res. Pap. 100, 117–126. (doi:10.1016/j.dsr.2015.02.013)

[22] Smith PK et al. 1985 Measurement of protein using bicinchoninic acid. Anal. Biochem. 150, 76–85. (doi:10.1016/0003-2697(85)90442-7) 3843705 10.1016/0003-2697(85)90442-7

[23] Bligh E, Dyer W 1959 A rapid method of total lipid extraction and purification. Can. J. Biochem. Physiol. 37, 911 (doi:10.1139/y59-099) 13671378 10.1139/o59-099

[24] Marsh J, Weinstein D 1966 Simple charring method for determination of lipids. J. Lipid Res. 7, 574–576. 5965305

[25] Dubois M, Gilles K, Hamilton J, Rebers P, Smith F 1956 Colorimetric method for determination of sugars and related substances. Anal. Chem. 28, 350–356. (doi:10.1021/ac60111a017)

[26] Froese R, Pauly D 2015 FishBase [WWW Document]. *World Wide Web Electron. Publ*. See www.fishbase.org.

[27] Felsenstein J 1985 1956 Phylogenies and the comparative method. Am. Nat. 125, 1–15. (doi:10.1086/284325)

[28] R Core Development Team. 2015 R: a language and environment for statistical computing. Vienna, Austria: R Foundation for Statistical Computing.

[29] Bailey D, Bagley P, Jamieson A, Collins M, Priede I 2003 *In situ* investigation of burst swimming and muscle performance in the deep-sea fish *Antimora rostrata* (Günther, 1878). J. Exp. Mar. Biol. Ecol. 286, 295–311. (doi:10.1016/S0022-0981(02)00534-8)

[30] Collins MA, Priede IG, Bagley PM 1999 *In situ* comparison of activity in two deep-sea scavenging fishes occupying different depth zones. Proc. R. Soc. Lond. B 266, 2011–2016. (doi:10.1098/rspb.1999.0879)

[31] Kenaley CP, Stote A, Flammang BE 2014 The morphological basis of labriform rowing in the deep-sea bigscale *Scopelogadus beanii* (Percomorpha: Beryciformes). J. Exp. Mar. Bio. Ecol. 461, 297–305. (doi:10.1016/j.jembe.2014.07.024)

[32] Luck DG, Pietsch TW 2008 Observations of a deep-sea ceratioid anglerfish of the genus *Oneirodes* (Lophiiformes: Oneirodidae). Copeia 2008, 446–451. (doi:10.1643/CE-07-075)

[33] Lauder GV, Flammang B, Alben S 2012 Passive robotic models of propulsion by the bodies and caudal fins of fish. Integr. Comp. Biol. 52, 576–587. (doi:10.1093/icb/ics096) 22740513 10.1093/icb/ics096

[34] Leftwich M, Tytell E, Cohen A, Smits A 2012 Wake structures behind a swimming robotic lamprey with a passively flexible tail. J. Exp. Biol. 215, 416–425. (doi:10.1242/jeb.061440) 22246250 10.1242/jeb.061440 PMC3257170

[35] Tangorra J, Phelan C, Esposito C, Lauder G 2011 Use of biorobotic models of highly deformable fins for studying the mechanics and control of fin forces in fishes. Integr. Comp. Biol. 51, 176–189. (doi:10.1093/icb/icr036) 21653544 10.1093/icb/icr036

[36] Fujii T, Jamieson A, Solan M, Bagley P, Priede I 2010 A large aggregation of liparids at 7703 meters and a reappraisal of the abundance and diversity of hadal fish. Bioscience 60, 506–515. (doi:10.1525/bio.2010.60.7.6)

[37] Schneider CA, Rasband WS, Eliceiri KW 2012 NIH image to ImageJ: 25 years of image analysis. Nat. Methods 9, 671–675. (doi:10.1038/nmeth.2089) 22930834 10.1038/nmeth.2089 PMC5554542

[38] Chen S 2002 Fauna Sinica, Osteichthyes. Myctophiformes, Cetomimiformes, Osteoglossiformes. *Fauna Sinica Series*, 349p Beijing, People's Republic of China: Science Press.

[39] Endo H, Okamura O 1992 New records of the abyssal grenadiers *Coryphaenoides armatus* and *C. yaquinae* from the western North Pacific. Jpn. J. Ichthyol. 38, 433–437.

[40] Jamieson AJ, Fujii T, Solan M, Matsumoto AK, Bagley PM, Priede IG 2009 Liparid and macrourid fishes of the hadal zone: *in situ* observations of activity and feeding behaviour. Proc. R. Soc. B 276, 1037–1045. (doi:10.1098/rspb.2008.1670) 10.1098/rspb.2008.1670 PMC2679086 19129104

[41] Pietsch TW 1986 Melanocetidae. In Smith's sea fishes (eds Smith MM, Heemstra PC), pp. 375–376. Berlin, Germany: Springer-Verlag.

[42] Møller PR, Knudsen SW, Schwarzhans W, Nielsen JG 2016 A new classification of viviparous brotulas (Bythitidae) – with family status for Dinematichthyidae – based on molecular, morphological, and fossil data. Mol. Phylogenet. Evol. 100, 391–408. (doi:10.1016/j.ympev.2016.04.008) 27060424 10.1016/j.ympev.2016.04.008

[43] Nielsen JG 1998 Encyclopedia of fishes (ed. Paxton JR, Eschmeyer), WN p. 134 San Diego, CA: Academic Press (ISBN 0-12-547665-5)

[44] Nielsen JG, Cohen DM, Markle DF, Robins CR 1999 Ophidiiform fishes of the world (order Ophidiiformes): an annotated and illustrated catalogue of pearlfishes, cusk-eels, brotulas and other ophidiiform fishes known to date. FAO Fish. Synop. 125, 178p.

[45] Nielsen JG 1986 Ophidiidae. In Fishes of the North-eastern Atlantic and the Mediterranean, vol. 3 (eds Whitehead PJP, Bauchot M-L, Hureau J-C, Nielsen J, Tortonese E), pp. 1158--1166. Paris, France: UNESCO.

[46] Bianchi G, Carpenter KE, Roux J-P, Molloy FJ, Boyer D, Boyer HJ 1999 Field guide to the living marine resources of Namibia. *FAO species identification guide for fishery purposes*, 265 p. Rome, Italy: FAO.

[47] Russian Academy of Sciences. 2000 *Catalog of vertebrates of Kamchatka and adjacent waters*. 166 p.

[48] Shinohara G, Yabe K, Nakaya M, Anma G, Yamaguchi S, Amaoka K 1994 Deep-sea fishes collected from the North Pacific by the T/S Oshoro-Maru. Bull. Fac. Fish. Hokkaido Univ. 45, 48–80.

[49] Fedorov VV, Chereshnev IA, Nazarkin MV, Shestakov AV, Volobuev VV 2003 Catalog of marine and freshwater fishes of the northern part of the Sea of Okhotsk, 204 p Vladivostok, Russia: Dalnauka.

[50] Anderson ME 1994 Systematics and osteology of the Zoarcidae (Teleostei: Perciformes), 120 p Grahamstown, South Africa: Ichthyol. Bull. J.L.B. Smith Inst. Ichthyol.

[51] Anderson M, Hubbs C 1981 Redescription and osteology of the North-eastern Pacific fish *Derepodichthys alepidotus* (*Zoarcidae*). Copeia 1981, 341–352. (doi:10.2307/1444223)

[52] Hart JL 1973 *Pacific fishes of Canada*, 180, 740 p. Fisheries Research Board of Canada.

[53] Peden AE 1979 Meristic variation of Lycodapus mandibularis (Pisces: Zoarcidae) and oceanic upwelling on the west coast of North America. J. Fish. Res. Board Can. 36, 69--76. (doi:10.1139/f79-009)

[54] Anderson ME 2012 A new species of *Pachycara* Zugmayer (Teleostei: Zoarcidae) from off Monterey Bay, California, USA, with comments on two North Pacific *Lycenchelys* species. Zootaxa 3559, 39–43.

[55] Vetter R, Lynn E, Costa A, Garza M 1994 Depth zonation and metabolic adaptation in Dover sole, *Microstomus pacificus*, and other deep-living flatfishes: factors that affect the sole. Mar. Biol. 120, 145–159.

[56] Friedman J, Condon N, Drazen J 2012 Gill surface area and metabolic enzyme activities of demersal fishes associated with the oxygen minimum zone off California. Limnol. Oceanogr. 57, 1701–1710. (doi:10.4319/lo.2012.57.6.1701)

[57] Masuda HK, Amaoka K, Araga C, Uyeno T, Yoshino T 1984 The fishes of the Japanese archipelago, vol. 1 437 p Tokyo, Japan Tokai University Press.

[58] Parin NV, Fedorov VV, Sheiko BA 2002 An annotated catalogue of fish-like vertebrates and fishes of the seas of Russia and adjacent countries: part 2. Order Scorpaeniformes. J. Ichthyol. 42(Suppl.1), S60–S135.

[59] Andriashev AP 1998 A review of recent studies of Southern Ocean Liparidae (Teleostei: Scorpaeniformes). Cybium 22, 255–266.

[60] Andriashev A, Stein D 1998 Review of the snailfish genus *Careproctus* (Liparidae, Scorpaeniformes) in Antarctic and adjacent waters. *Nat. Hist. Museum Los Angeles Cty* Contrib. Sci. 470, 1–63.

[61] Kai Y, Orr J, Sakai K, Nakabo T 2011 Genetic and morphological evidence for cryptic diversity in the *Careproctus rastrinus* species complex (Liparidae) of the North Pacific. Ichthyol. Res. 58, 143–154. (doi:10.1007/s10228-010-0202-2)

[62] Knudsen SW, Møller PR 2008 *Careproctus kidoi*, a new Arctic species of snailfish (Teleostei: Liparidae) from Baffin Bay. Ichthyol. Res. 55, 175–182. (doi:10.1007/s10228-007-0034-x)

[63] Stein DL 2005 Descriptions of four new species, redescription of *Paraliparis membranaceus*, and additional data on species of the fish family Liparidae (Pisces, Scorpaeniformes) from the west coast of South America and the Indian Ocean. Zootaxa 1019, 1–25. (doi:10.11646/zootaxa.1019.1.1)

[64] Linley TD, Gerringer ME, Yancey PH, Drazen JC, Weinstock CL, Jamieson AJ 2016 Fishes of the hadal zone including new species, *in situ* observations and depth records of Liparidae. Deep Sea Res. I. 114, 99–110. (doi:10.106/j.dsr.2016.05.003)

[65] Busby M, Cartwright R 2009 *Paraliparis adustus* and *Paraliparis bullacephalus*: two new snailfish species (Teleostei: Liparidae) from Alaska. Ichthyol. Res. 56, 245–252. (doi:10.1007/s10228-008-0090-x)

[66] Chernova N, Møller P 2008 A new snailfish, *Paraliparis nigellus* sp. nov. (Scorpaeniformes, Liparidae), from the northern Mid-Atlantic Ridge – with notes on occurrence of *Psednos* in the area. Mar. Biol. Res. 4, 369–375. (doi:10.1080/17451000802017507)

[67] Stein DL, Chernova N, Andriashev AP 2001 Snailfishes (Pisces: Liparidae) of Australia, including descriptions of thirty new species. Rec. Aust. Museum 53, 341–406. (doi:10.3853/j.0067-1975.53.2001.1351)

[68] Chernova N 2001 A review of the genus *Psednos* (Pisces, Liparidae) with description of ten new species from the north Atlantic and southwestern Indian Ocean. Bull. Mus. Comp. Zool. 155, 477–507.

[69] Andriashev AP, Pitruk DL 1993 A review of the ultra-abyssal (hadal) genus *Pseudoliparis* (Scorpaeniformes, Liparidae) with a description of a new species from the Japan Trench. Vopr. Iktiologii 33, 325–330.

[70] Gerringer ME, Linley TD, Jamieson AJ, Goetze E, Drazen JC 2017 *Pseudoliparis swirei* sp. nov.: a newly-discovered hadal snailfish (Scorpaeniformes: Liparidae) from the Mariana Trench. Zootaxa. 4358, 161–177. (doi:10.11646/zootaxa.4358.1.7) 29245485 10.11646/zootaxa.4358.1.7

[71] Nielsen JG 1990 Ophidiidae. In Check-list of the fishes of the eastern tropical atlantic (CLOFETA), vol. 2 (eds Quero JC, Hureau JC, Karrer C, Post A, Saldanha L), pp. 564--573. Lisbon, Portugal: JNICT, SEI and UNESCO.

[72] Yancey P, Gerringer M, Drazen J, Rowden A, Jamieson A 2014 Marine fish may be biochemically constrained from inhabiting the deepest ocean depths. Proc. Natl Acad. Sci. USA 111, 4461–4465. (doi:10.1073/pnas.1322003111) 24591588 10.1073/pnas.1322003111 PMC3970477

[73] Maddison W, FitzJohn R 2015 The unsolved challenge to phylogenetic correlation tests for categorical characters. Syst. Biol. 64, 127–136. (doi:10.1093/sysbio/syu070) 25209222 10.1093/sysbio/syu070

[74] Near TJ et al. 2013 Phylogeny and tempo of diversification in the superradiation of spiny-rayed fishes. Proc. Natl Acad. Sci USA 110, 12 738–12 743. (doi:10.1073/pnas.1304661110) 10.1073/pnas.1304661110 PMC3732986 23858462

[75] Betancur-R R et al. 2013 The tree of life and a new classification of bony fishes. PLoS Curr. Tree Life Edition 1 (doi:10.1371/currents.tol.53ba26640df0ccaee75bb165c8c26288) 10.1371/currents.tol.53ba26640df0ccaee75bb165c8c26288 PMC3644299 23653398

[76] Tsukamoto K et al. 2009 Positive buoyancy in eel leptocephali: an adaptation for life in the ocean surface layer. Mar. Biol. 156, 835–846. (doi:10.1007/s00227-008-1123-8)

[77] Drazen JC 2007 Depth related trends in proximate composition of demersal fishes in the eastern North Pacific. Deep Sea Res. Part I. Oceanogr. Res. Pap. 54, 203–219. (doi:10.1016/j.dsr.2006.10.007)

[78] Long JH Jr, Porter ME, Root RG, Liaw CW 2010 Go reconfigure: how fish change shape as they swim and evolve. Integr. Comp. Biol. 50, 1120--1139. (doi:10.1093/icb/icq066) 21558263 10.1093/icb/icq066

[79] Tytell ED, Leftwich MC, Hsu C-Y, Griffith BD, Cohen AH, Smits AJ, Hamlet C, Fauci LJ 2016 Role of body stiffness in undulatory swimming: insights from robotic and computational models. Phys. Rev. Fluids 1, 073202 (doi:10.1103/PhysRevFluids.1.073202)

[80] McHenry MJ, Pell CA, Long JH Jr 1995 Mechanical control of swimming speed: stiffness and axial wave form in undulating fish models. J. Exp. Biol. 198, 2293–2305. 9320209 10.1242/jeb.198.11.2293

