# Supplementary material for: Distribution, composition and functions of gelatinous tissues in deep-sea fishes
Source: R Soc Open Sci. 2017 Dec 6;4(12):171063. doi: 10.1098/rsos.171063 (PMC5750012; doi:10.1098/rsos.171063)
Supplement: Supplementary Table 1 [file rsos171063supp1.pdf]

Distribution, composition, and functions of gelatinous tissues in deep-sea fishes

Gerringer, Mackenzie E., Drazen, Jeffrey C., Linley, Thomas D., Summers, Adam P., Jamieson, Alan J., Yancey, Paul H.

Supplementary Table 1. Specimen information for gelatinous tissue samples tested. *N. kermadecensis* specimens were collected by free-vehicle trap (described Jamieson et al., 2013). Other specimens were collected by trawl (Drazen et al. 2015). Capture depth in metres. Collection dates noted. Standard length (SL) and Total length (TL) presented in centimetres, mass in grams.

Family	Species	Depth	Date	SL	TL	Mass	Sex
Liparidae	*Careproctus cypselurus*	1000	4.8.09		17.8	47.8	M
	Careproctus melanurus	750	10.2.09	15.5		24.47	F
		750	10.2.09	13.7	15.2	36.46	F
		1000	4.8.09		25.6	193.2	F
	Notoliparis kermadecensis	7000	11.29.11	24.5			
		7000	11.29.11	22.9			F
		7200	5.4.14	25.9	28	230	F
		7392	5.2.14	16.7	18.5	76	M
		7515	5.3.14	20.9	22.9	104	F
Ophidiidae	*Spectrunculus grandis*	2000	10.9.09	141	146	17463.3	
		4149	4.20.14	70	73.8	2128	F
Pleuronectidae	*Embassichthys bathybius*	1000	10.1.09	32.4	37.4	627.4	M
		1000	10.10.09	31.7	35	735.1	F
		1000	10.11.09	37.5	41.4	880.3	F
		1000	10.11.09	30.2	33.7	537.4	
	Microstomus pacificus	1000	10.1.09	46.6	50.4	1342.3	F
		1000	10.1.09	40.3	46.2	1127.1	F
		1000	10.1.09	43.7	48.5	1224.8	F
Zoarcidae	*Bothrocara brunneum*	1000	10.11.09	51	52.9	707	M
		2000	4.13.09	57.7	59.3	890.1	F
		2000	4.13.09		59.5	779.7	F
	Pachycara karenae	3000	10.8.09	35.6	37.4	503.6	F
		3000	10.8.09	34.7	36.6	450.8	M
		3000	10.8.09	37.7	38.5	501.6	F
	Pyrolycus sp.	4817	4.16.14	42	43.4	508	F
